# Non-toxic polymer nanovectors for improved delivery of dexamethasone

**DOI:** 10.1038/s41598-021-96797-4

**Published:** 2021-08-26

**Authors:** Benjamin C. Ede, Paraskevi Diamanti, David S. Williams, Allison Blair

**Affiliations:** 1 School of Cellular and Molecular Medicine, University of Bristol, University Walk, Bristol, BS8 1TD UK; 2 Bristol Institute for Transfusion Sciences, NHS Blood and Transplant Filton, Bristol, UK

**Keywords:** Drug delivery, Acute lymphocytic leukaemia, Drug delivery

## Abstract

Dexamethasone (Dex) is a highly insoluble front-line drug used in cancer therapy. Data from clinical trials indicates that the pharmacokinetics of Dex vary considerably between patients and prolonging drug exposure rather than increasing absolute dose may improve efficacy. Non-toxic, fully biodegradable Dex loaded nanovectors (NV) were formulated, via simple direct hydration within 10 min, as a vehicle to extend exposure and distribution in vivo. Dex-NV were just as effective as the free drug against primary human leukemia cells in vitro and in vivo. Importantly, high levels of DMSO solvent were not required in the NV formulations. Broad distribution of NV was seen rapidly following inoculation into mice. NV accumulated in major organs, including bone marrow and brain, known sanctuary sites for ALL. The study describes a non-toxic, more easily scalable system for improving Dex solubility for use in cancer and can be applied to other medical conditions associated with inflammation.

## Introduction

In developed countries, survival rates for childhood T-cell acute lymphoblastic leukemia (T-ALL) have increased to over 85%. Despite this, a considerable proportion of patients fail to respond to initial treatment or relapse with disease after reaching remission^1^. Furthermore, drug related side-effects can be severe, leading to treatment related morbidities and, in some cases, death^2^. Dexamethasone (Dex) is a highly insoluble, glucocorticoid used in front line therapy for several cancers. Dex is currently receiving considerable worldwide attention following a recent report that it reduced the deaths in critically ill patients with severe acute respiratory syndrome coronavirus 2 (SARS-CoV-2) from 41 to 29%^3^. However, its use is associated with severe adverse effects, including increased risk of developing life-threatening infections^2^ and osteonecrosis^4^. Furthermore due to its instability, Dex requires chemical modification into a salt form (Dexamethasone sodium phosphate) to be used intravenously. Developing methods to improve Dex solubility, without using toxic solvents, should improve its efficacy and reduce the associated adverse effects.

Clinical trials in cancer, have attempted to modify Dex doses to alleviate side-effects whilst maintaining efficacy^5^. Although there were no significant differences in responses at day 28, interestingly, a 12-fold range in Dex exposure was reported following administration of a single dose and exposure was higher in patients with a low blast count (< 5%) at day 8^5^. This large variability in Dex clearance could be due to factors, such as age (as younger patients have been shown to clear Dex faster)^6^, expression of genes and proteins involved in drug efflux^7^, and reactions to other drugs such as asparaginase^6^. Higher rates of Dex clearance have previously been shown to correlate with higher rates of relapse, including in the central nervous system^8^. Together these data suggest that controlled exposure to Dex, rather than absolute dose, may be key to improving outcomes. Consequently, developing methods to extend Dex circulation and distribution may be fundamental to improving its therapeutic performance against cancers and pertinently, against SARS-CoV-2.

Achieving controlled drug release is a central ambition of the nanomedicine field, which seeks to develop nanoscopic carriers to improve drug performance by enhancing their stability, increasing solubility or directing activity to the desired cells/tissue^9^. Although examples of such nanomedicines abound in the literature, it is important to select a suitable carrier system with ease-of-use, biocompatibility and good structural characterisation, since the goal is to reduce off-target toxicity and improve overall drug utility. Carrier-based approaches have been tested for the delivery of Dex to enhance drug bioavailability for cancers^10–12^, cardiovascular and neurological diseases^13^ and inflammation^14^. Unfortunately, in previous studies the problem of carrier toxicity or scalability presented significant barriers to further development.

**Figure 1:**
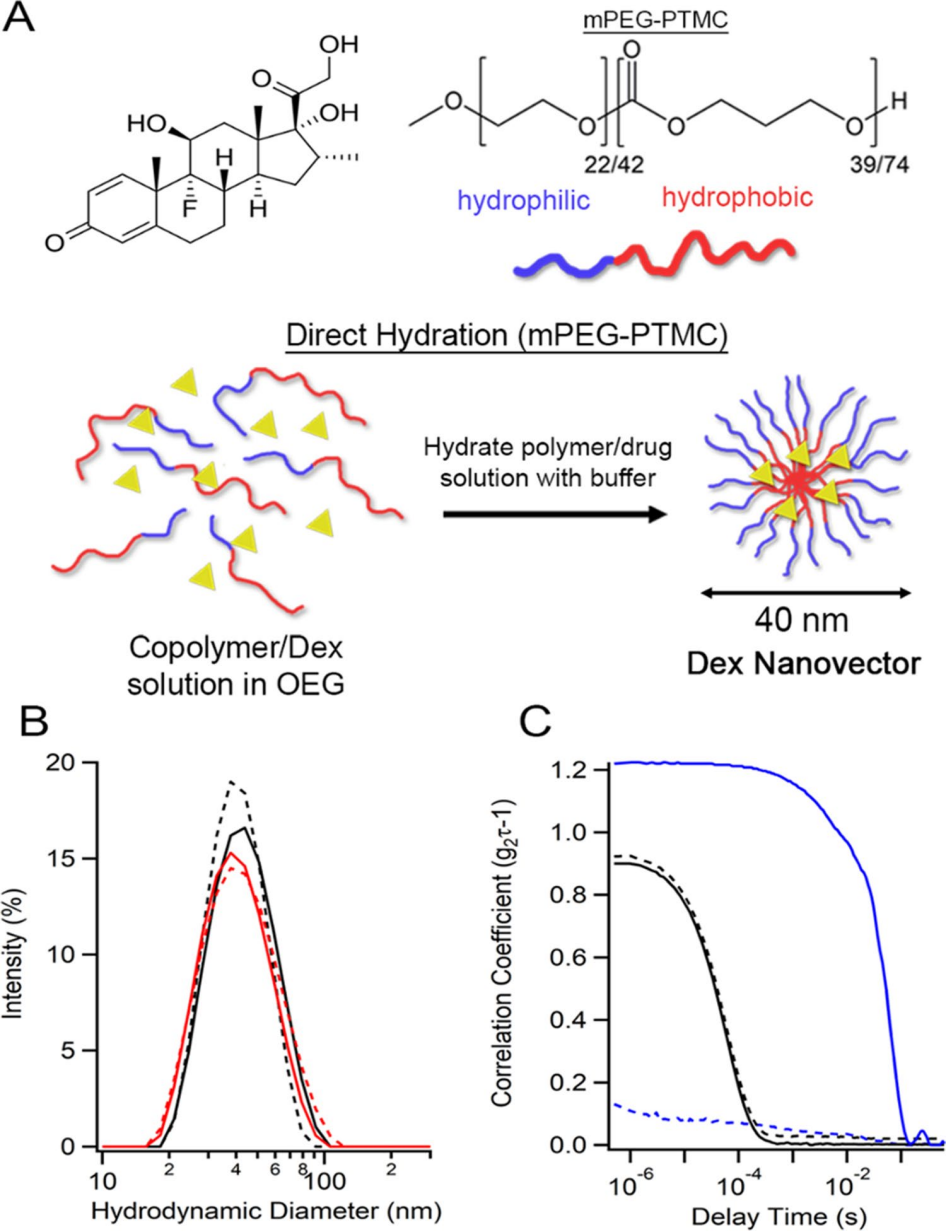
Fabrication of Dex-loaded Nanovectors (Dex-NV). (**A**) Schematic outlining the fabrication of Dex-NV comprising mPEG-PTMC copolymers. (**B**) DLS size measurements of NV loaded with Dex (Black) or DiR (Red) before (solid) and after (dashed lines) 0.2 µm filtration. (**C**) DLS correlogram data of NV (black) or free Dex (blue), before (solid) and after (dashed lines) filtration. *Dex* dexamethasone, *DiR* 1,1′-dioctadecyl-3,3,3′,3′-tetramethylindotricarbocyanine iodide, *DLS* dynamic light scattering.

More recently, our group have developed drug-loaded nanovectors (NV) comprising copolymer amphiphiles based upon biocompatible poly(ethylene glycol) (mPEG) and poly(trimethylene carbonate) (PTMC) that undergo spontaneous self-assembly from drug-containing oligo(ethylene glycol) (OEG) solution upon addition of buffer (a process called direct hydration, Fig. 1A)^11^. Furthermore, we demonstrated the non-toxicity and therapeutic potential of this mPEG-PTMC NV platform to treat primary paediatric ALL samples in vitro. In the present study we have utilised this platform to demonstrate NV’s potential for improved delivery of Dex against primary childhood T-ALL in vivo*,* without the use of harmful levels of solvents.

## Results and discussion

Using an optimised protocol for the direct hydration of NV, it was established that the upper limit of Dex encapsulation was 5 wt% with 100% loading efficiency, confirmed using dynamic light scattering (DLS). Therapeutically relevant concentrations of Dex (0.25–0.5 mg/mL) giving 2.5–5 mg/kg doses in mice (equivalent to 5–10 mg/m^2^ in humans) were encapsulated, which contained ≤ 0.5% DMSO to enhance its dissolution during the direct hydration process (Fig. 1A). The prepared 5 wt% Dex-NV had an average size of 41 ± 1 nm and narrow polydispersity index (< 0.14), which did not significantly change after 0.2 µm filtration (Fig. 1B). Without NV, free Dex solutions contained large, heterogeneous precipitates that were almost entirely removed by filtration (Fig. 1C, Fig. S1). The presence of Dex in NV formulations, after filtration, was confirmed by the elevated 242 nm absorbance signal (Figure S1). There was no significant difference in the critical micelle concentration (CMC) between Dex-NV and empty NV, with NV forming stably above 0.025 mg/mL copolymer (Figure S1), well below the concentration that would be reached in vivo in this study.

**Figure 2:**
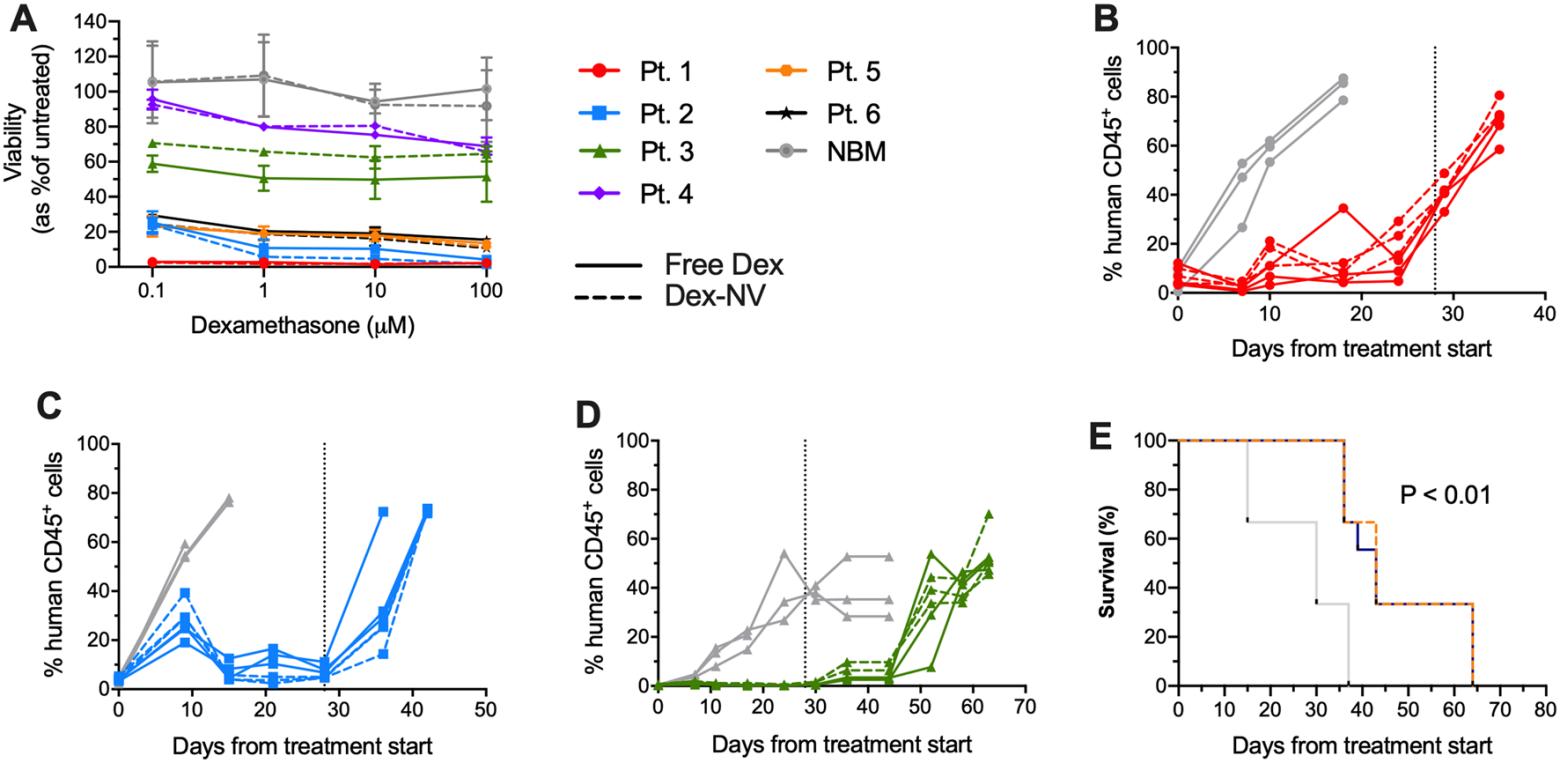
Efficacy of Dex-NV in vitro and in vivo. (**A**) Viability of T-ALL samples (pts. 1–6) and normal bone marrow (NBM, n = 3) treated with Dex-NV (dashed lines) or free Dex (solid lines) for 48 h. Viability was assessed by flow cytometry using Annexin-V and PI, expressed as a proportion of the untreated control sample. Data represent mean ± SD of at least 2 replicate measurements. (**B**-**D**) T-ALL cells from pts. 1–3, respectively, were inoculated into NSG mice and the levels of human cells (CD45^+^ and CD7^+^) were measured in weekly PB aspirates. Once engraftment reached > 0.1%, mice were treated IP with Dex-NV (dashed coloured line), free Dex (solid coloured line) both at 2.5 mg/kg or placebo (grey line) 5 times per week over 28 days. Engraftment levels of individual mice (n = 3 per group) are shown. Vertical dotted lines indicate end of Dex treatment. (**E**) Kaplan Meier survival analysis of ALL engrafted NSG (n = 9 per group), treated with Dex-NV (dashed line), free Dex (solid line) or placebo (grey line) IP. Survival data analysed by Log-rank test. **P < 0.01. *IP* intraperitoneal, *NV* nanovectors, *PI* propidium iodide.

Treating primary T-ALL samples with Dex-NV revealed this system was equipotent to free Dex (dispersed in medium from DMSO stock solution at 0.5 mg/mL). The sensitivity to Dex, varied between the 6 samples tested, with pts. 1, 2, 5 and 6 being very sensitive, < 16% cell survival at the highest dose tested (100 μM) with half maximal inhibitory concentration, (IC_50_)_,_ < 100 nM. In contrast, pts. 3 and 4 were resistant, both failing to reach an IC_50_ value (Fig. 2A). Superior leukaemia cell killing was observed in pt. 6 using Dex-NV compared to free drug (P < 0.04). Results in normal bone marrow controls did not differ between the two formulations. Crucially, these data demonstrate that NV formulation did not reduce Dex efficacy and confirmed our previous findings using parthenolide in this system^11^. This represents a major advantage over other carriers, such as Pluronic F-127, where drug performance can be reduced following encapsulation^15^.

For in vivo studies, cells from 3 of these cases were inoculated into NOD.Cg-Prkdc^scid^Il2rγ^tm1Wjl^/SzJ (NSG) mice. Once ≥ 0.1% human CD45^+^ cells were detected in peripheral blood (PB) aspirates, separate cohorts of animals were treated with Dex-NV, free Dex (IP, 2.5 mg/kg,) or placebo daily, for 5 days/week over a 4 week period. Both Dex-NV and free Dex delayed disease progression until day 18 in pt.1 (Fig. 2B) and until after treatment cessation in pts. 2 and 3 (Fig. 2C,D). In contrast, disease progression was rapid in all control animals (Fig. 2B–D). Dex-NV and free Dex significantly improved the survival of NSG by up to 27 days (P < 0.01, Fig. 2E). Dex-NV administered IV (5 mg/kg, 3 days/week for 4 weeks), was also equipotent to free Dex (Figure S2) and there were no differences in NV efficacy using IP or IV administration. Importantly empty NV and drug loaded had no adverse effects on healthy animals, regardless of administration route (Figure S3).

In a comparable study, Krishnan et al. used Dex-loaded mPEG-poly(caprolactone) (PEG-PCL) particles (fabricated by nanoprecipitation) to treat mice engrafted with an adult ALL cell line (RS4;11) and demonstrated improved survival by 3 days compared to free Dex^12^. However, PEG-PCL particles, unlike mPEG-PTMC used here, necessitate greater efforts in particle fabrication and purification, with greatly reduced encapsulation efficiency. In contrast, Dex-NV, used in this report, were prepared in 10 min via simple direct hydration (with very low levels of organic solvent) and have now been shown to effectively treat primary T-ALL in vivo, without loss of efficacy.

**Figure 3:**
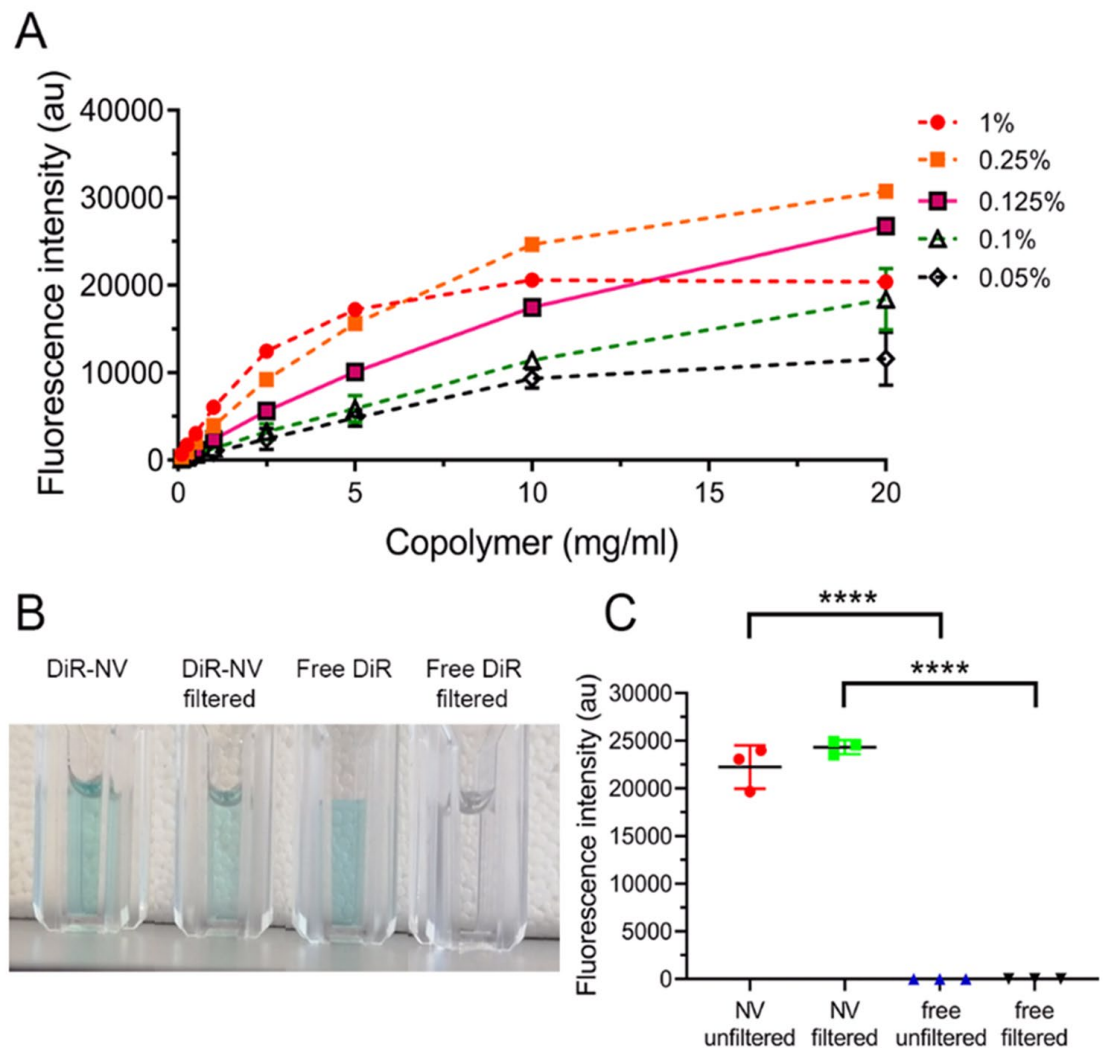
Formation of DiR loaded NV. (**A**) Fluorescence intensity of different wt% loadings of DiR in NV at different concentrations of copolymer (0.1–20 mg/mL) in PBS. Solid line indicates the most linear increase in fluorescence intensity (0.125 wt% loading DiR). (**B**) Macroscopic images of DiR-NV and free DiR, pre and post filtration (0.2 μm). (**C**) Fluorescence intensity of 0.125 wt% DiR-NV and free DiR pre and post filtration. Fluorescence intensity data represents mean ± SD. ****P < 0.0001. *DiR* 1,1′dioctadecyl 3,3,3′,3′-tetramethylindotricarbocyanine iodide, *PBS* phosphate buffered saline.

As stated above, extending the circulation time and distribution of Dex may be key to improving outcomes. To study distribution in vivo, fluorescent NV were prepared loaded with the lipophilic dye 1,1′-Dioctadecyl-3,3,3′,3′-tetramethylindotricarbocyanine iodide (DiR) that only fluoresces in hydrophobic microenvironments, such as lipid bilayers and the NV core. Following the seminal work of Meng et al., the loading content of DiR within NV was optimised to ensure linear concentration dependence and avoid any subsequent inaccuracies due to fluorophore de-quenching^16^. DiR-NV were prepared with a range of wt% of fluorophore (0.05–1) and tested over a wide range of copolymer concentrations (0.1-20 mg/mL), with 0.125 wt% giving the best linearity (r^2^ = 0.97, Fig. 3A). Without being stabilised by NV, DiR aggregated and was removed by 0.2 µm filtration, as indicated by the loss of solution colour (Fig. 3B) and DLS data (Figure S4). Filtration of DiR-NV did not change fluorescence intensity, whereas free DiR in solution (aggregates) showed no fluorescent signal (Fig. 3C). Furthermore, DiR-NV were identical to their Dex counterpart in terms of size (Fig. 1B). Cells treated with DiR-NV showed a steady increase in fluorescence for up to 8 h, suggesting controlled cellular endocytosis and uptake (Figure S5), in agreement with our previous study^11^.

**Figure 4:**
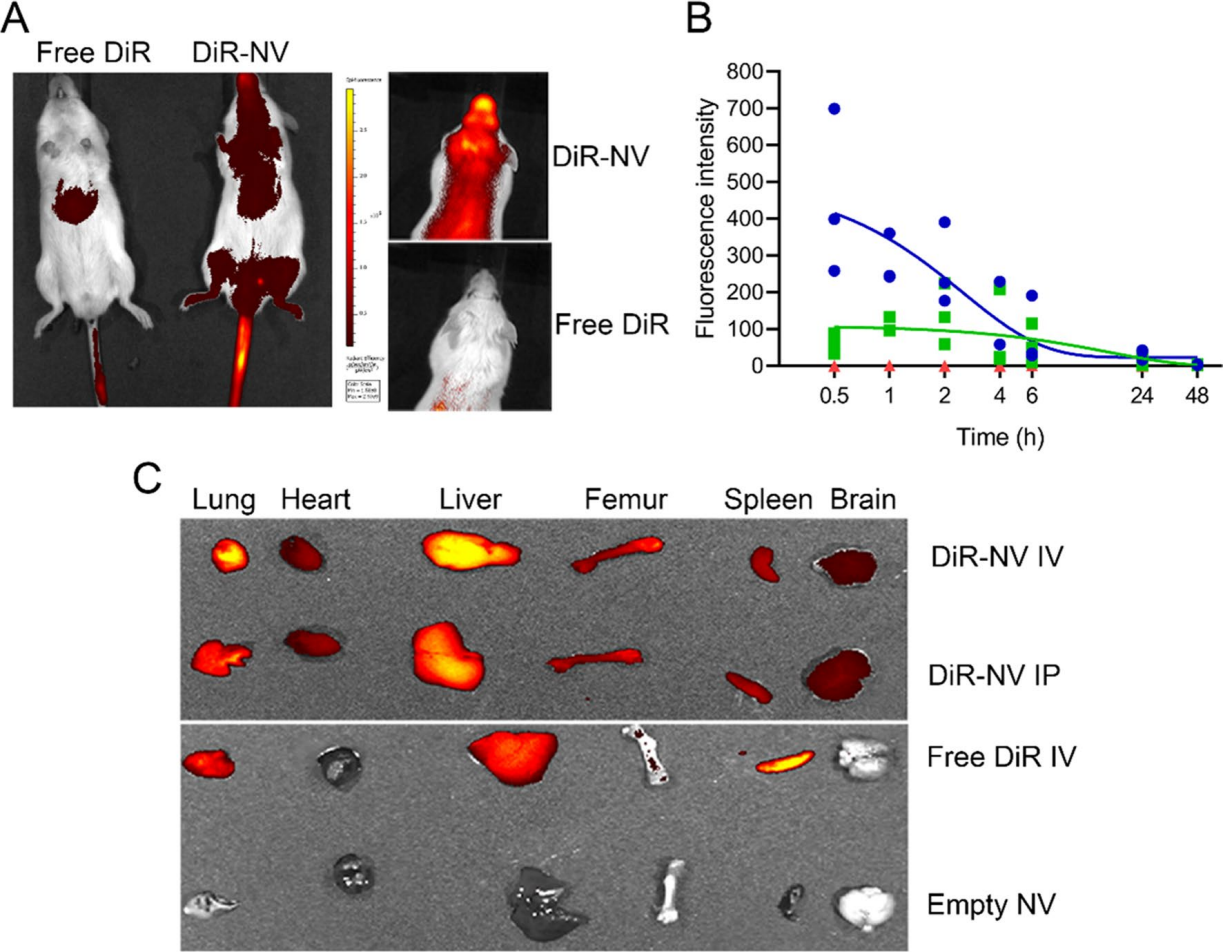
Distribution of DiR-loaded NV. (**A**) In vivo ventral and dorsal fluorescence imaging (radiant efficiency × 10^8^) obtained from IVIS of live NSG mice treated with 0.125 wt% DiR-NV or free DiR for 45 min. (**B**) Fluorescence intensity of tail vein bleeds from NSG mice treated with 0.125 wt% DiR-NV IV (blue, n = 3), IP (green, n = 3) or free drug IV (red, n = 3). Solid lines represent one phase exponential decay of IV treated NSG. (**C**) IVIS fluorescence images of organs harvested from NSG mice 24 h after treatment with 0.125 wt% DiR-NV (IV and IP), free DiR or empty NV. *NSG* NOD.Cg-Prkdc^scid^Il2rγ^tm1Wjl^/SzJ, *IV* intravenous, *IVIS* in vivo imaging system, *IP* intraperitoneal.

Ten minutes after IV injection, DiR-NV fluorescence was detected throughout NSG mice using ventral imaging (Fig. 4A). Fluorescence was also detected in PB aspirated from the opposite tail vein to that injected, from the first time point (452 ± 225 RFU, 30 min), proving evidence of NV circulation (Fig. 4B). The half-life of DiR-NV in PB was 2.25 h for IV treated mice. As would be expected, peak fluorescence took longer in IP treated mice (2 h, 138 ± 83 RFU) and had a half-life of 3.2 h from peak fluorescence. Fluorescence remained undetectable in free DiR treated mice over the 48-h period. Dorsal imaging also showed accumulation of DiR-NV in the head area, indicative of uniquely broad distribution properties (Fig. 4A). In contrast, free DiR, used as a low solubility control, accumulated in the liver over the 45 min of imaging and showed very low levels in the PB (Fig. 4A,B). After 24 h, NV accumulated in the major organs including lungs, liver and spleen, regardless of administration route (Fig. 4C). Interestingly, significant accumulations in the bone marrow (BM) and brain were also observed, which are known sanctuary sites for childhood ALL. Mononuclear cells from femoral BM, harvested from mice 24 h after treatment with DiR-NV, had increased median fluorescence intensity (676) compared to cells from animals treated with free DiR (366) or empty NV (284), confirming the presence of NV in BM cavities (Figure S6). Due to the small size of NV used in this study (40 nm diameter) they are well-suited to permeate diverse biological layers that can impede larger particles. Indeed, pegylated nanoparticles < 100 nm are known to display reduced hepatic filtration, longer circulation time and a high rate of extravasation into permeable tissues^17^. Importantly, we have recently shown that NV encapsulation results in more controlled release of Dex compared to the free drug, confirming the advantages of this system^18^.

In conclusion, this study provides proof of principle that mPEG-PTMC NV can be loaded with Dex at high concentrations, using the direct hydration process, and are effective against ALL in vivo. This NV system is easily prepared, stable for several months and does not require extensive purification, as compared to traditional formulation methods. Importantly, it has been demonstrated that NV circulate to sanctuary sites, including the BM and CNS where increased drug exposure is likely to enhance overall drug efficacy. In addition, NV can cross the blood brain barrier, so this system has the potential to treat the emerging neurological issues in SARS-CoV-2 pts. Future work will aim to enhance NV targeting to sanctuary sites to improve efficacy and minimise off-target toxicity, and to explore the role of morphology upon the circulation/distribution of NV in vivo*.*

## Methods

### PEG–PTMC block copolymer synthesis

Poly ethylene glycol block poly trimethylene carbonate (PEG–PTMC) copolymers were synthesized as previously reported^11^. Briefly, monomethoxy PEG-OH (Mw = 1 kDa, Đ < 1.1, Rapp Polymers, Tübingen, Germany) was combined with a stoichiometric amount of trimethylene carbonate (3.8 mmol, Thermo Fisher Scientific, Loughborough, UK) to synthesize copolymers comprising 20 wt% PEG. Dry toluene (≈ 50 mL) was then added and evaporated to ensure dryness. To initiate polymerization, dry dichloromethane (DCM, 10 mL) and methanesulfonic acid (0.1 mmol = 6 µL) were added under argon and stirred for 24 h at 30 °C. The reaction mixture was then mixed with DCM, washed with saturated NaHCO_3_, followed by brine and dried over Na_2_SO_4_, concentrated and precipitated into ice cold diethyl ether (≈ 100 mL, all Sigma-Aldrich, Gillingham, UK). The solid was re-dissolved in dioxane and lyophilised.

### Drug encapsulation in nanovectors via direct hydration

PEG-PTMC copolymer was dissolved in oligo(ethylene glycol) (OEG, Sigma-Aldrich) at 20 wt% at 45 °C and mixed to ensure homogeneity. To form micelles, 10 µL of PEG-PTMC solution was added to a 1.5 mL Eppendorf and vortexed for 1 min with buffer to the desired concentration of copolymer. Dex or 1,1′-dioctadecyl-3,3,3′,3′-tetramethylindotricarbocyanine iodide (DiR, Perkin Elmer, Llantrisant, UK) encapsulation was achieved by dissolving dry drug to 100 mg/mL in dimethyl sulfoxide (DMSO, Origen Biomedical, Austin, USA). A small volume of drug solution was mixed with the copolymer OEG solution to achieve the desired wt% loading of drug, without the need for purification.

### Nanovector stability

The stability of NV was assessed using DLS. NV were analysed using a Malvern Instruments Zetasizer Nano (ZSP). Zetasizer software (Malvern Panalytical, Malvern, UK) was used for data analysis. CMC were determined by adding 10 µM of 3,3′-dioctadecyloxacarbocyanine Perchlorate (DiO), which fluoresces in hydrophobic conditions, to different concentrations of NV copolymer (0.00125–0.5 mg/mL). DiO fluorescence was detected using a SynergyNeo2 plate reader (BioTek, Swindon, UK).

### Spectral scanning of Dex in solution

Dex-NV and free Dex preparations (0.125 mg/mL) were analysed in a UV-STAR MICROPLATE (Greiner Bio One, Stonehouse, UK). Absorption spectroscopy was performed from 230-500 nm using a SynergyNeo2 plate reader (BioTek, Swindon, UK).

### Human samples

Bone marrow samples from 5 children and a young adult diagnosed with T-ALL at disease presentation were used in this study (Supplemental Table 1). Normal bone marrow samples from healthy individuals were used as controls. All samples were collected with informed consent from the individuals or patients or their parents/guardians. T-ALL samples were supplied by Blood Cancer UK Childhood Leukaemia Cell Bank. The study was approved by the University Hospitals Bristol and Weston NHS Foundation Trust and London Brent Research Ethics Committee and all methods were conducted in accordance with the relevant guidelines and regulations. Mononuclear cells were harvested via density gradient centrifugation using Ficoll-Hypaque (Sigma-Aldrich) and suspended in 90% fetal calf serum (FCS, Thermo Fisher Scientific) and 10% dimethyl sulfoxide (DMSO) and stored in liquid nitrogen prior to use.

### Drug toxicity assay

ALL cells were plated at 2 × 10^5^ per mL in RPMI-1640 media supplemented with 20% FCS, 1% (vol/vol) l-glutamine and 1% (vol/vol) penicillin (100 U/mL) streptomycin (100 μg/mL) (all Thermo Fisher Scientific) and treated with Dex-NV or free Dex. Cells were incubated with drug for 48 h at 37 °C in a humidified atmosphere with 5% O_2_/CO_2_. Cells were then resuspended in 100 μL annexin-V buffer consisting of 10 mM HEPES, 150 mM NaCl and 2.5 mM CaCl_2_ (all Sigma-Aldrich) dissolved in PBS at pH 7.4 and stained with a 1:20 dilution of annexin-V conjugated to fluorescein isothiocyanate (Miltenyi Biotec, Bisley, UK). Cells were then washed, resuspended in annexin-V buffer, and stained with propidium iodide (PI, 1 µg/mL, Miltenyi Biotec) immediately prior to flow cytometric analysis on a MACsQuant flow cytometer (Miltenyi Biotec). Flow cytometry data was analysed using FlowJo analysis software version 10.0.8 (FlowJo, Ashland, USA).

### In vivo evaluation

Animal experiments were conducted in the University of Bristol Animal Services Unit, using procedures licensed and approved by the United Kingdom Home Office. All experiments were performed in accordance with Home Office regulations and reported in accordance with ARRIVE guidelines. Human T-ALL cells were injected via the lateral tail vein of 5–8 week old NSG mice and allowed to engraft. Human cell levels were measured by flow cytometric analysis of PB aspirates weekly, using anti-CD45 (BD Biosciences, Bisley UK). Once human CD45^+^ cells reached ≥ 0.1%, Dex-NV, free Dex or placebo was administered to separate groups of mice (n = 3/group) 5 days/week (2.5 mg/kg, IP) or 3 days/week (5 mg/kg, IV), for 4 weeks. Engraftment levels were monitored weekly and mice were maintained for up to 9 weeks or until they became symptomatic of the disease. NSG were killed, gross anatomy was inspected and femoral BM was assessed by flow cytometry for human cell engraftment using antibodies against human CD45 and CD7 (BD Biosciences). Major organs were collected for fluorescence imaging.

### Fluorescence intensity of DiR in vivo

NSG were inoculated IV with unencapsulated DiR (free DiR), empty NV, Free DiR or DiR-NV (n = 3/group). At specified time points, PB was collected into 10 µL heparin solution (360 units/mL, StemCell Technologies, Cambridge, UK) from the opposite tail vein to that used for inoculation. Fluorescence intensity of DiR was measured using a SynergyNeo2 plate reader with excitation and emission wavelengths of 710 and 760 nm, respectively. Separate cohorts of mice, treated as above, were terminated after 24 h and the florescence intensity of femoral BM mononuclear cells was measured by flow cytometry.

### Imaging analyses

Animals were anaesthetised using 2.5% isoflurane gas for up to 45 min and imaged using an IVIS Spectrum (Perkin Elmer) at excitation and emission wavelengths of 710 and 760 nm, respectively. Harvested organs were washed in PBS and imaged immediately.

### Statistical analyses

One-way analysis of variance followed by Tukey post hoc testing was used to compare fluorescence intensity and polydispersity index values for NV and free drug solutions. Survival data from in vivo experiments were analysed using Kaplan Meier survival analysis using log-rank test of survival distribution. Half-life of DiR loaded NV in NSG was calculated using one phase exponential decay analysis.

## Supplementary Information

Supplementary Information 1.
