# Supplementary material for: Non-toxic polymer nanovectors for improved delivery of dexamethasone
Source: Sci Rep. 2021 Aug 26;11:17263. doi: 10.1038/s41598-021-96797-4 (PMC8390661; doi:10.1038/s41598-021-96797-4)
Supplement: Supplementary file 1 — Supplementary Information 1. [file 41598_2021_96797_MOESM1_ESM.pdf]

Supplemental Data

Non-toxic polymer nanovectors for improved delivery of dexamethasone

Benjamin C. Ede, Paraskevi Diamanti, David S. Williams and Allison Blair

Supplemental Table 1 Patient characteristics

Patient ID	Karyotype	Age at diagnosis (y)	Gender	MRD risk status
1	t(11;14)	2	M	Low
2	t(1;14)	3	M	Low
3	ABL1	10	M	Low
4	+9	6	M	Low
5	del 6	13	M	Risk
6	14q11	21	M	N/A

Low and risk MRD status was defined at day 29. N/A, not available.

Supplemental Table 2 **Percentage of viable T-ALL cells following treatment with free Dex or Dex-NV (Fig. 2A)**

Patient ID	1	2	3	4	5	6
**Untreated**	66.1±2.1	24.7±10.2	12.8±0.5	49.0±1.9	40.9±3.1	20.8±2.7
**Free Dex (0.1μM)**	1.9±0.8	4.0±1	6.5±0.9	47.0±2.7	9.5±2.4	6.4±0.5
**Free Dex (1μM)**	1.8±1.1	1.7±0.8	6.3±1.4	39.1±1.1	7.8±1.7	4.4±0.6
**Free Dex (10μM)**	0.9±0.1	1.6±0.2	6.6±1.8	36.9±0.6	7.4±1.3	4.1±0.8
**Free Dex (100μM)**	1.5±0.4	0.7±0.1	6.7±1.3	33.8±2.4	5.5±0.4	3.9±0.4
**Dex-NV (0.1μM)**	1.7±0.1	8.0±1.4	8.2±0.3	45.2±1.4	8.9±2.8	4.9±0.3
**Dex-NV (1μM)**	1.1±0.1	1.9±0/1	7.8±0.8	39.1±1.1	7.1±0.4	3.8±0.5
**Dex-NV (10μM)**	1.3±0.2	1.6±0.1	8.1±0.6	39.3±0.6	6.4±0.4	3.3±0.8
**Dex-NV (100μM)**	1.3±0.4	0.5±0.1	8.5±0.2	32.1±0.5	4.3±0.4	2.2±0.2

Data represents mean±SD from at least 2 replicates for each dose.

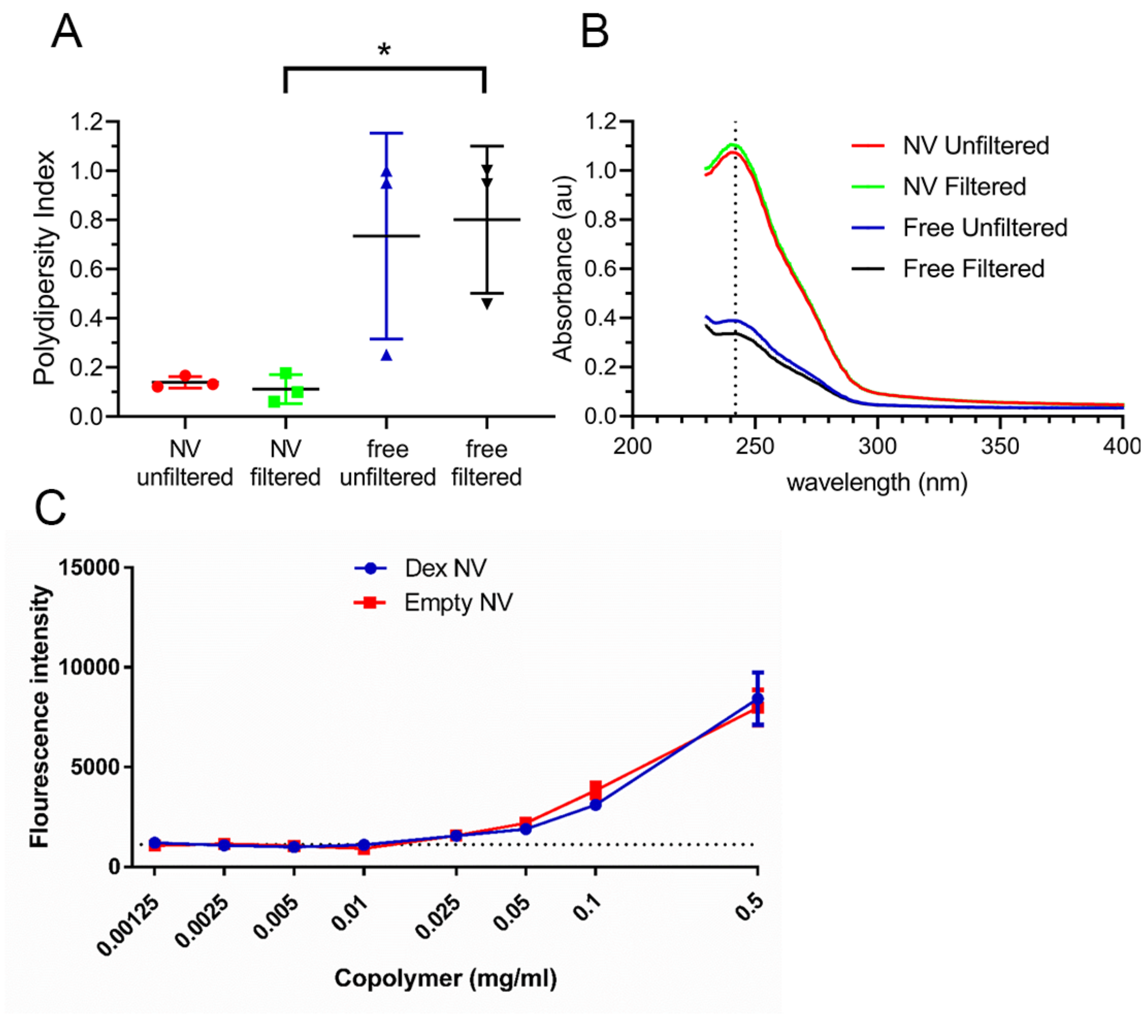

Figure S1 Formation of Dex loaded PEG-PTMC NV

A) Polydispersity index (PDI) values of Dex-NV formulations and free Dex from DLS pre and post filtration (200nM). B) Spectral scan of Dex-NV formulations and free drug, the dotted line indicates the expected wavelength of Dex absorbance (242nm) in solution. C) Critical micelle concentration determination of Dex-NV (blue) or empty NV (red) using 10µM DiO over different concentrations of copolymer (0.00125-0.5 mg/mL) in PBS. PDI data represented as mean±SD (n=3). Fluorescence intensity data represented as mean±SD (n=3). *$P < 0.05$.

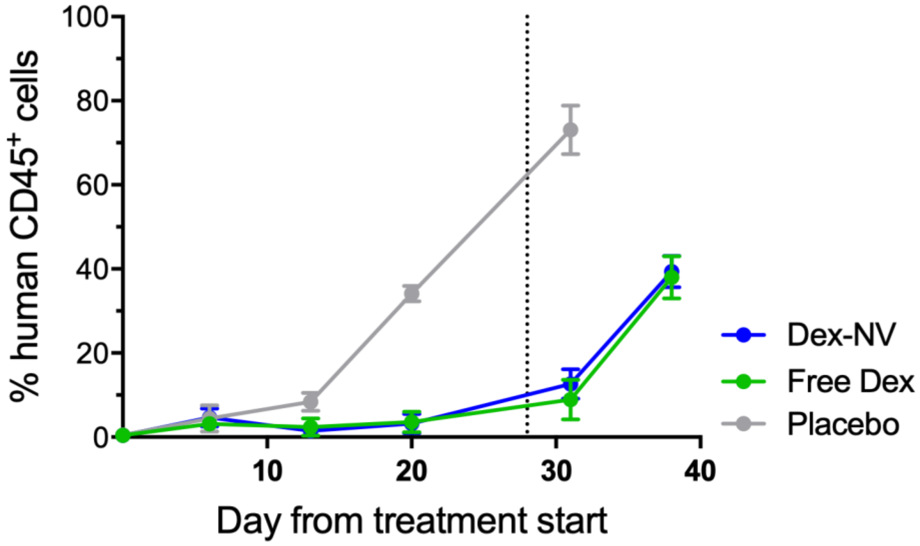

Figure S2 Dex-NV IV treatment efficacy

Cells from pt.1 were inoculated into NSG mice. Human cell ($CD45^+$ and $CD7^+$) levels were measured in peripheral blood (PB) weekly. Once engraftment reached >0.1%, mice were treated IV with Dex-NV (Blue), free Dex (Green) both at 5mg/kg or placebo (Grey) 3 times per week over a 4-week period. Lines represent proportion of human cells detected in PB aspirates from individual mice (n=3 per treatment arm). Vertical dotted line indicates end of treatment.

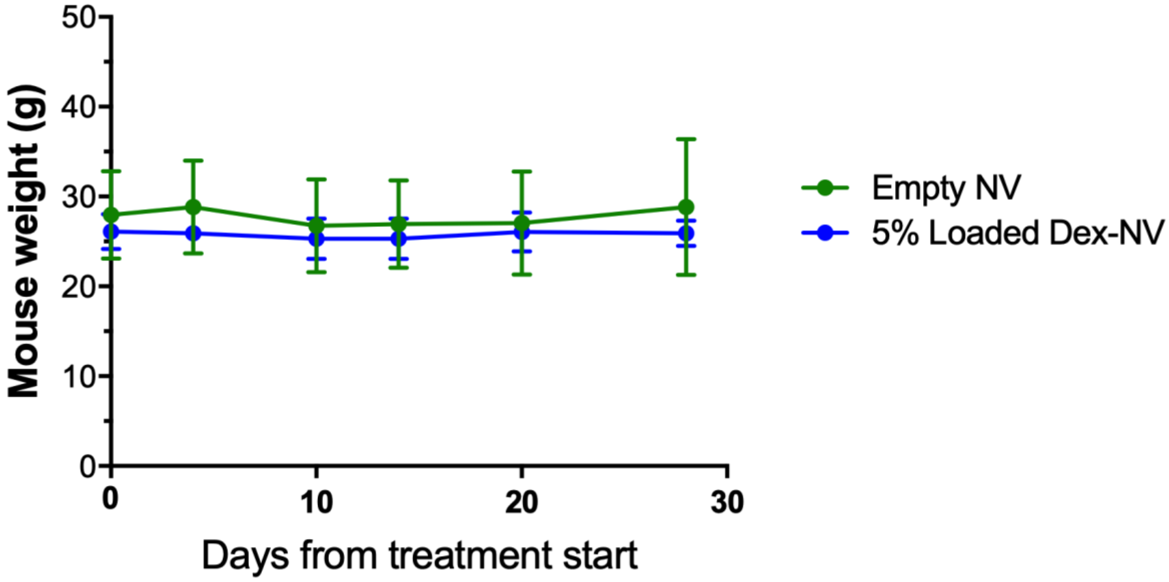

Figure S3 Effect of empty and Dex-loaded NV on NSG body weight

NSG mice were treated ip with empty NV or 5% loaded Dex-NV daily for 5 days over a 4-week period. Lines represent mean body weights of up to 3 mice, error bars represent SD.

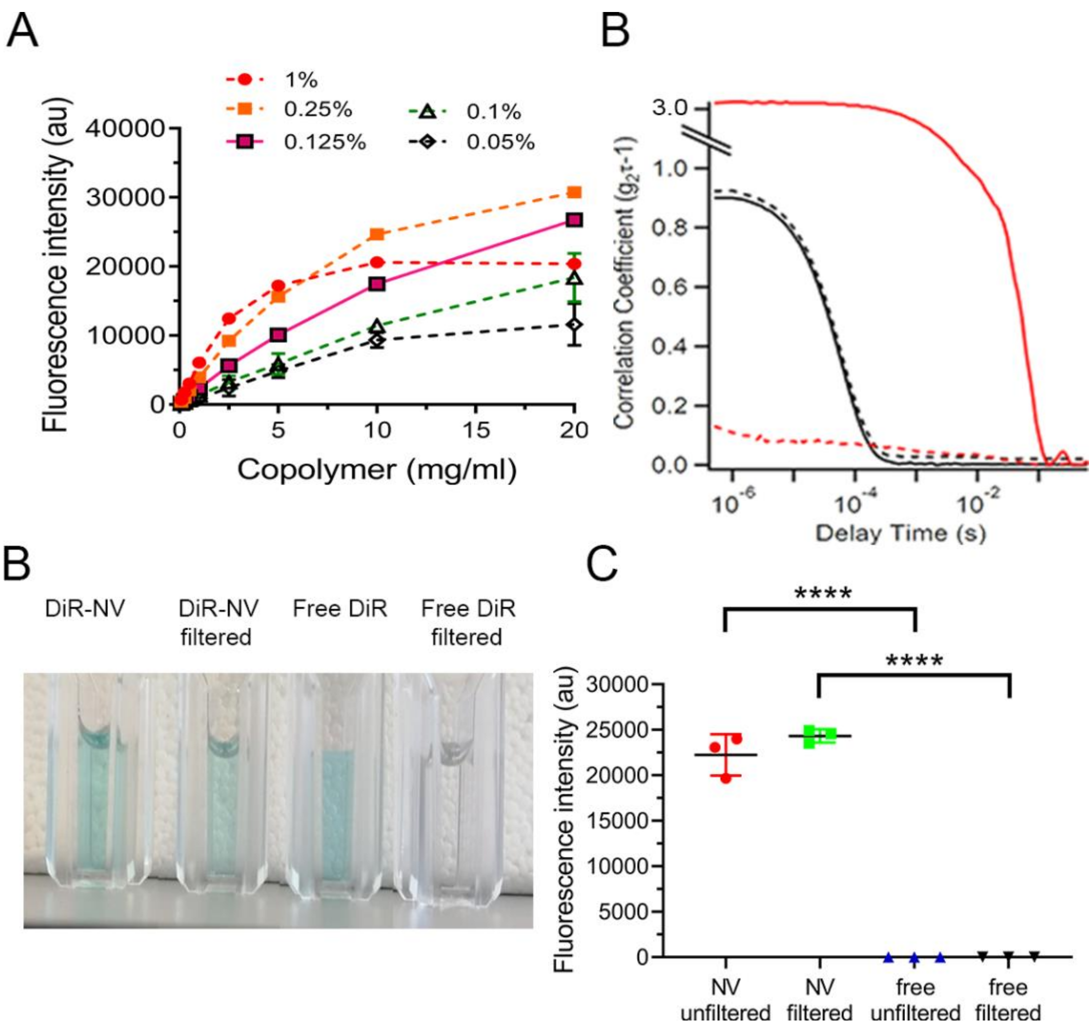

Figure S4 **Formation of DiR loaded PEG-PTMC NV**

A) Fluorescence intensity of different wt% loadings of DiR in NV at different concentrations of copolymer (0.1-20 mg/mL) in PBS. Solid line indicates the most linear increase in fluorescence intensity (0.125 wt% loading DiR). B) DLS correlogram data of NV (black) or free DiR (red), before (solid) and after (dashed lines) filtration. Fluorescence intensity data represented as mean±SD (n=3). C) Macroscopic images of DiR-NV and free DiR, pre and post filtration (0.2µm). D) Fluorescence intensity of 0.125 wt% DiR-NV and free DiR pre and post filtration. Fluorescence intensity data represents mean±SD. ****P < 0.001. DiR,1,1'Dioctadecyl 3,3,3',3'-Tetramethylindotricarbocyanine Iodide; DLS, dynamic light scattering; PBS, phosphate buffered saline.

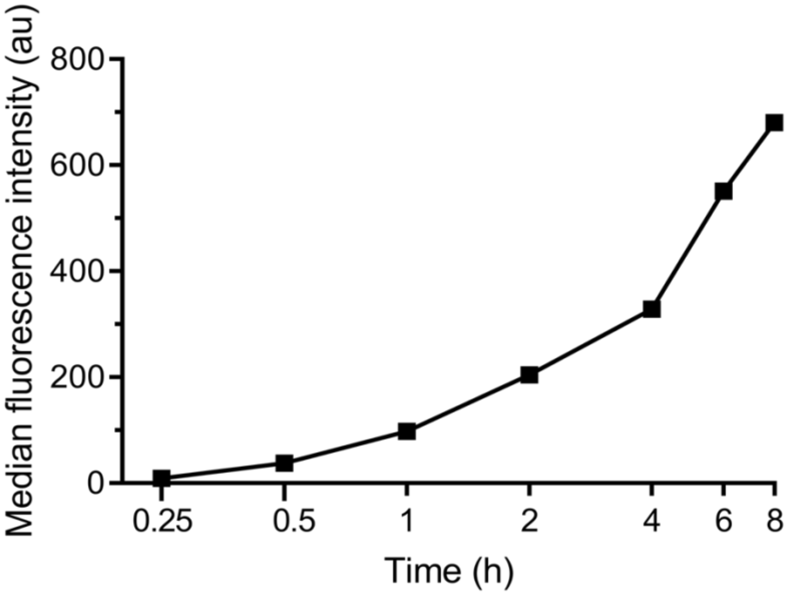

Figure S5 Accumulation of DiR-NV in ALL cells

Median fluorescence intensity of T-ALL cells from pt.1 treated with 0.125 wt% DiR-NV for 8 hours. Fluorescence intensity was determined by flow cytometry in viable (propidium iodide negative) cells.

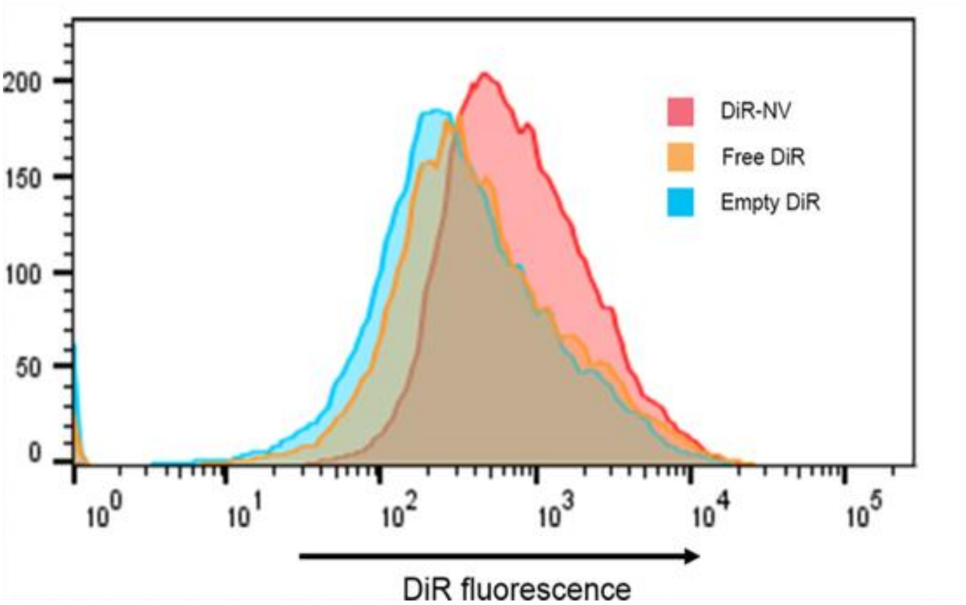

Figure S6 Accumulation of DiR-NV in NSG BM cells

Histograms representing the fluorescence intensity of DiR in mononuclear BM cells derived from NSG mice 24 hours after IV administration of DiR-NV (red), free DiR (orange) or empty NV (blue). Fluorescence intensity was determined by flow cytometry.
